# The effectiveness of community health worker training, equipping, and deployment in reducing COVID-19 infections and deaths in rural Western Kenya: A comparison of two counties

**DOI:** 10.1371/journal.pgph.0003036

**Published:** 2024-03-25

**Authors:** Neema Kaseje, Kennedy Oruenjo, Dan Kaseje, Meghna Ranganathan, Marcel Tanner, Andy Haines

**Affiliations:** 1 Surgical Systems Research Group, Kisumu, Kenya; 2 London School of Hygiene & Tropical Medicine, London, United Kingdom; 3 Siaya Ministry of Health, Siaya, Kenya; 4 Tropical Institute of Community Health, Kisumu, Kenya; 5 Swiss Tropical & Public Health Institute, Basel, Switzerland; PLOS: Public Library of Science, UNITED STATES

## Abstract

COVID-19 and other pandemics remain significant threats to population health, particularly in rural settings where health systems are disproportionately weak. There is a lack of evidence on whether trained, equipped, and deployed community health workers (CHWs) can lead to significant reductions in COVID-19 infections and deaths. Our objective was to measure the effectiveness of deploying trained and equipped CHWs in reducing COVID-19 infections and deaths by comparing outcomes in two counties in rural Western Kenya, a setting with limited critical care capacity and limited access to COVID-19 vaccines and oral COVID-19 antivirals. In Siaya, trained CHWs equipped with thermometers, pulse oximeters, and KN95 masks, visited households to convey health information about COVID-19 prevention. They screened, isolated, and referred COVID-19 cases to facilities with oxygen capacity. They measured and digitally recorded vital signs at the household level. In Kisii county, the standard Kenya national COVID-19 protocol was implemented. We performed a comparative analysis of differences in CHW skills, activity, and COVID-19 infections and deaths using district health information system (DHIS2) data. Trained Siaya CHWs were more skilled in using pulse oximeters and digitally reporting vital signs at the household level. The mean number of oxygen saturation measurements conducted in Siaya was 24.19 per COVID-19 infection; and the mean number of temperature measurements per COVID-19 infection was 17.08. Siaya CHWs conducted significantly more household visits than Kisii CHWs (the mean monthly CHW household visits in Siaya was 146,648.5, standard deviation 11,066.5 versus 42,644.5 in Kisii, standard deviation 899.5, p value = 0.01). Deploying trained and equipped CHWs in rural Western Kenya was associated with lower risk ratios for COVID-19 infections and deaths: 0.54, 95% CI [0.48–0.61] and 0.29, CI [0.13–0.65], respectively, consistent with a beneficial effect.

## Introduction

The COVID-19 pandemic has had a significant impact on the health of populations across the globe. As of January 2024, globally, there have been more than 774,291,287 million infections and up to 18 million deaths directly and indirectly linked to COVID-19 [1]. In sub-Saharan Africa (SSA), as of January 2024, 9,571,930 million COVID-19 infections were confirmed and 175,486 deaths were recorded [2]. The pandemic response was a challenge in sub-Saharan Africa (SSA) because of under-resourced health systems with limited access to critical care capacity, COVID-19 vaccines and COVID-19 therapeutics [3]. Moreover, over 50% of the population in SSA lives in rural areas, with under-resourced rural health systems that are more vulnerable to disruptions during pandemics and poorer access to health services compared with urban health systems [4, 5].

In the current literature, there is evidence to support the key role CHWs can play during pandemics including the COVID-19 pandemic [6]. Qualitative data from India, Bangladesh, Pakistan, Sierra Leone, Kenya and Ethiopia, document the important role CHWs played in surveillance, community education and support of community members with COVID-19 [7]. An assessment of CHW preparedness in Kenya, Senegal, and Uganda by Chengo et al. found, however, that CHWs faced significant challenges because of the lack of training and personal protective equipment (PPE) [8]. Other studies demonstrated that training, especially of rural CHWs, led to improved COVID-19 surveillance efforts and reduced COVID-19 infections. In rural Thailand, trained rural CHWs were effective in screening and referring suspected COVID-19 cases which limited the transmission of COVID-19 to rural parts of Thailand [9]. In rural Niger, trained CHWs reported valid COVID-19 alerts in 84% of cases [10].

**Fig 1:**
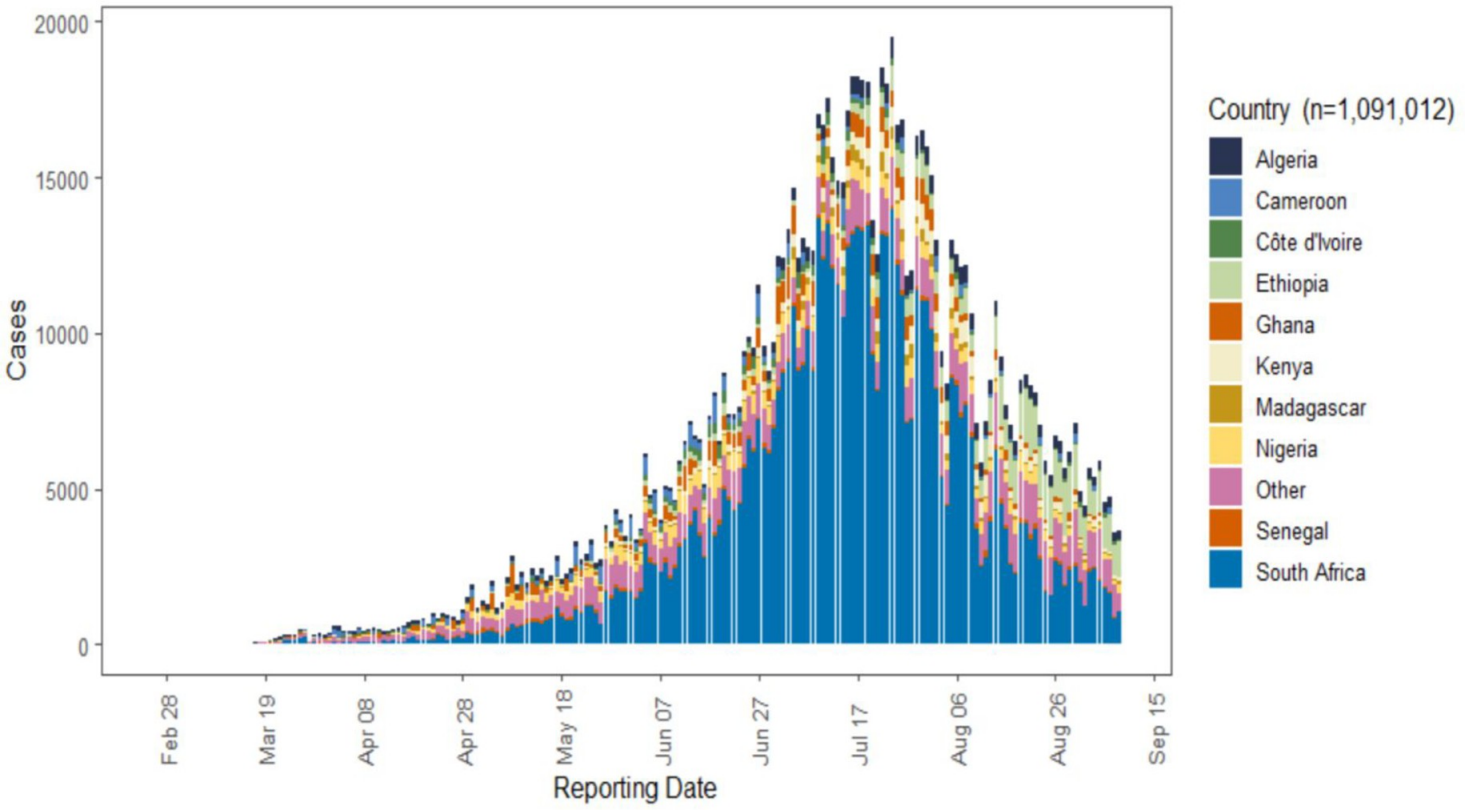
An epicurve of confirmed cases of COVID-19 in the WHO African Region 25 February—8 September 2020 n = 1 091 012 [6].

In line with global trends, SSA and Kenya experienced progressive increases in the number of COVID-19 cases during the early phase of the COVID-19 pandemic. The timeline of COVID-19 infections in SSA and Kenya, is shown in Fig 1 [11].

Additionally, there were differences in COVID-19 measures implemented across countries and in Kenya lockdown measures were implemented early during the COVID-19 pandemic. Below is a summary of the COVID-19 timeline and key measures that were taken to mitigate the impact of the COVID-19 pandemic in Kenya.

### COVID-19 timeline Kenya

Kenya reported its first case of COVID-19 on March 13th 2020 and immediately instituted mandatory quarantine for positive cases [12]. There were movement restrictions and curfews from April 2020 and lockdown measures in high-risk counties including Nairobi, Mombasa, Kilifi, and Kwale [12]. In addition, schools and universities were closed, and there were restrictions on social gatherings [12]. However, in July 2020, movement restrictions and lockdowns were lifted [12]. Kenya received its first batch of COVID-19 vaccines in March 2021 [12, 13].

Rural health systems in SSA face unique challenges [5, 14]. They are under-resourced because of a lack of adequate equipment, infrastructure, and health personnel [5, 14]. We confirmed these challenges in a baseline assessment of the Siaya rural health system conducted early during the COVID-19 response [15]. The baseline assessment demonstrated a limited workforce consisting mostly of nurses at the health facility level [15]. A cohort of community health workers (CHWs) active since the 1970s provided preventive and promotive health information to households [16]. They conformed to the 2018 WHO definition of CHWs: CHWs provide health education and referrals for a wide range of services, and provide support and assistance to communities, families and individuals with preventive health measures and gaining access to appropriate curative health and social services [17]. They create a bridge between providers of health, social and community services and communities that may have difficulty accessing these services [17]. In the Kenyan context, CHWs function within community units (CUs) each serving a population of 5000 people [18]. They are trained to increase demand for health services at the community level [18]. In Kenya, there are an estimated 6359 CUs and 63590 CHWs [18].

Early during the COVID-19 pandemic, we recognized the critical role CHWs could potentially play in the COVID-19 response, because they have the most frequent contact with households of all health workers, and they are trusted members of their communities [19–21]. As a result, they are well-positioned to address sensitive and stigmatizing issues including detection of active COVID-19 infections and the need to isolate active COVID-19 cases without instilling fear among community members or policing them. We therefore assessed CHW knowledge of COVID-19 and its case management in rural Western Kenya [15]. This baseline assessment showed that CHWs had no prior knowledge of COVID-19 and its case management; nor did they have prior experience of using thermometers and pulse oximeters or of digitally reporting vital signs at the household level [15].

Although the current evidence of the effectiveness of rural CHWs in improving health outcomes during the COVID-19 pandemic is suggestive, there are limitations in the research methodologies used to date and the outcome measures reported [9, 10, 22–26]. First, studies measuring the effectiveness of rural CHWs during the COVID-19 pandemic lacked comparative components in their research designs limiting conclusions about the strength of the causal link between building the capacity of CHWs and improved health outcomes during the COVID-19 pandemic [9, 10, 22–26]. Second, outcome measures included CHW knowledge, CHW household visits, CHW referrals of symptomatic cases, the incidence of COVID-19 infections, and the rate of valid COVID-19 alerts; however, none of the studies provided mortality data [9, 10, 22–26]. Lastly, none of the studies addressed rural CHWs and rural populations in Western Kenya [9, 10, 22–26].

Therefore, the objective of this study was to measure the effectiveness of deploying trained and equipped rural CHWs in reducing COVID-19 infections and deaths in rural Western Kenya using a comparative analysis of an intervention county (Siaya) and a non-intervention county (Kisii).

## Methods

### Ethics statement

We received ethical review approvals from the Jaramogi Oginga Odinga Teaching and Referral Hospital Ethics Review Committee, and the London School of Hygiene and Tropical Medicine Ethics Review Committee (**approval numbers IERC/JOOTR/219/20 and 27252**).

### Study design

This study is a comparative analysis of Siaya where CHWs were trained, equipped and deployed and Kisii where the CHW intervention did not take place. For our analyses, we used the DHIS2, an anonymized aggregated health information database following approval from the Siaya Ministry of Health. As we had no access to information that could identify individual participants in the DHIS2, informed consent was not required.

### Settings

Siaya is a rural county in Western Kenya with a population of 993,183. Siaya has 240 community health units (CHUs); with approximately 10 CHWs covering each CHU.

Kisii is also a rural county in Western Kenya with a population of 1,266,860. It has 291 CHUs, with approximately 10 CHWs covering each unit.

The standard Kenya national COVID-19 protocol did not include CHW training, equipping with pulse oximeters, and digital monitoring of vital signs at the household level.

### Participants

CHWs and households in Siaya and Kisii Western Kenya.

The intervention in Siaya sought to reach 2000 CHWs covering 200,000 households.

### Research question

What is the effectiveness of training, equipping, and deploying CHWs in addition to the standard Kenya national COVID-19 protocol in reducing COVID-19 infections and deaths in rural Western Kenya?

### Intervention in Siaya county

Under the leadership of Siaya Ministry of Health (MOH), the intervention focused on training, equipping, and deploying CHWs to reduce COVID-19 infections and deaths in Siaya. In line with the human and financial resources that were available, the intervention took place from August 2020 to November 2020.

#### A: Training and equipping CHWs in Siaya county

The training was conducted using the following sequence:

a) ***Raising awareness about COVID-19*, *and preventing and detecting COVID-19 cases***:
  CHWs were trained on: raising awareness about COVID-19; COVID-19 prevention (with universal mask-wearing, frequent handwashing, physical distancing, and principles of infection prevention and control (IPC)); screening of potential cases; and contact tracing of community members potentially exposed to suspected and confirmed COVID-19 cases.
b) ***Isolating COVID-19 cases*:**
  CHWs were also trained on how to isolate suspected and confirmed COVID-19 cases to minimize further community transmission.
c) ***Managing COVID-19 cases*:**
  CHWs were trained to manage minor and moderate COVID-19 cases at home. In addition, they were trained to recognize severe COVID-19 cases and refer severe COVID-19 cases to health facilities with oxygen capacity. To optimize their diagnostic, clinical monitoring, and referral capacities, CHWs were also trained on the use of contactless thermometers and pulse oximeters. This component of the CHW training covered i) the pathophysiology of COVID-19 infection and how it affects the lung leading to hypoxemia ii) how pulse oximeters measure the oxygen saturation of hemoglobin molecules iii) possible causes of spurious readings iv) normal and abnormal oxygen saturation rates v) the action that needs to be taken when oxygen saturation rates were below 90%.
d) ***Measuring and digitally reporting vital signs at the household level*:**
  CHWs were trained on how to measure body temperatures and oxygen saturation levels and digitally report vital signs at the household level using Commcare, and the Whatsapp platform when additional support from a clinician was needed. Furthermore, they were updated on facilities with oxygen capacity. Commcare is an open source mobile platform that supports frontline workers in low-resource communities (https://www.dimagi.com/commcare/).
e) ***Use of essential health services*, *providing mental health support*, *and leadership during the COVID-19 pandemic*:**
  CHWs were trained to ensure households continued to use essential health services including maternal and child health services, during the COVID-19 pandemic. In addition, CHWs received training in leadership skills and providing psychological first aid.

In addition to training Siaya CHWs, we equipped them with KN95 masks, thermometers, and pulse oximeters. They received internet data for the reporting of household level vital signs.

#### B: CHW deployment: CHW household visits

Following training and receipt of equipment, CHWs visited households to raise awareness about COVID-19 and educate households about COVID-19 prevention with physical distancing, mask wearing, and handwashing. CHWs ensured households had functional handwashing points. They identified and isolated any symptomatic household members and educated them about minimizing transmission within households. They measured body temperatures and oxygen saturation levels of suspected and confirmed COVID-19 cases and digitally reported household level vital signs using Commcare and Whatsapp platforms. They referred severe COVID-19 cases to health facilities with oxygen capacity. They counselled households on the importance of the continued use of maternal and child health services during the COVID-19 pandemic. In addition, they conducted psychological support as needed.

**Fig 2:**
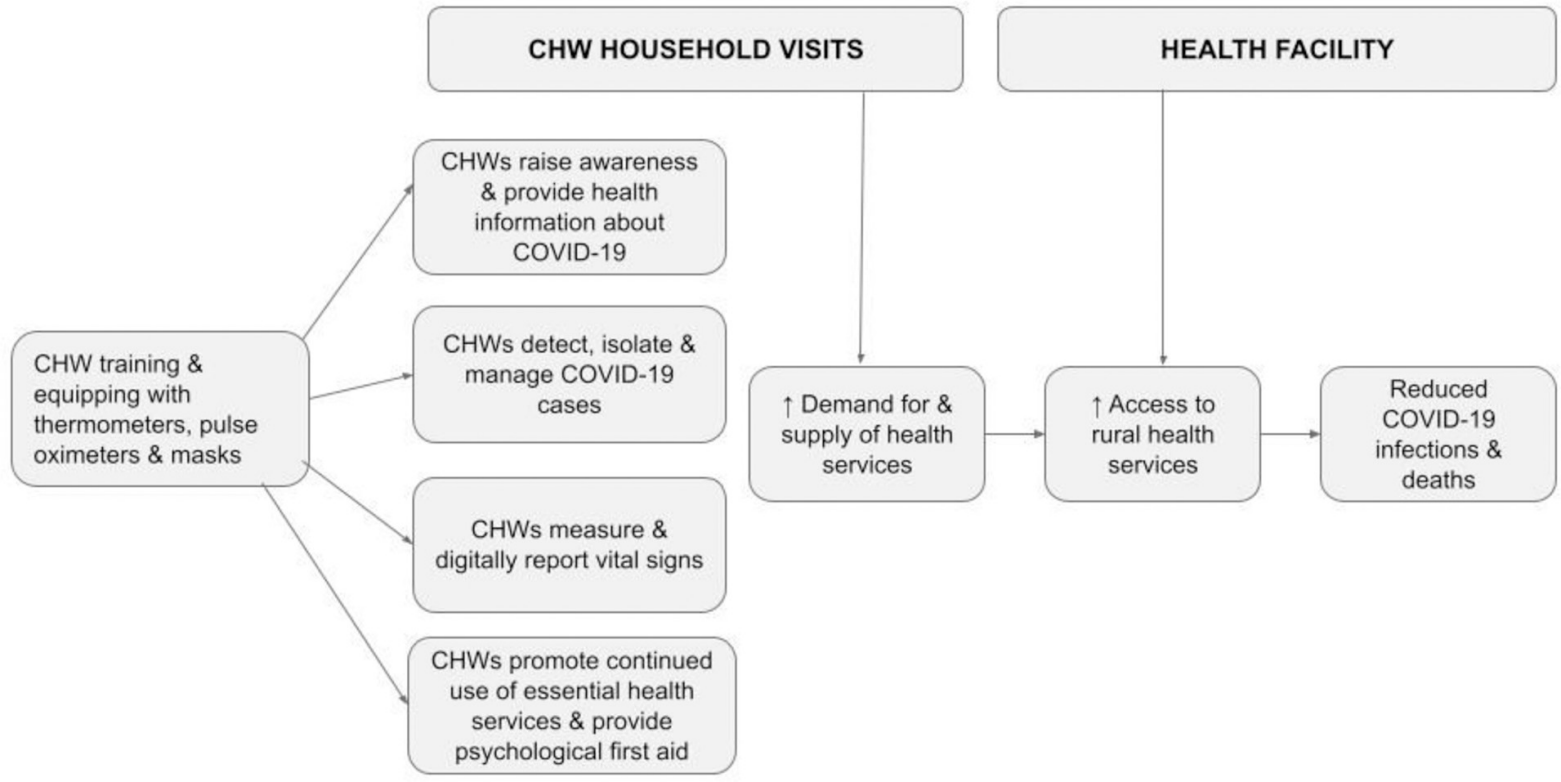
Training, equipping and deploying CHWs to reduce COVID-19 infections and deaths.

Fig 2 summarizes the intervention in Siaya.

For this study, we defined training as a process that provides conditions in which individuals gain knowledge, skills or ability [27]. In addition, equipping CHWs in this study meant the process by which CHWs had access to medical equipment including pulse oximeters and thermometers and digital tools for reporting vital signs. Additionally, CHWs were equipped with KN95 masks during a time when COVID-19 vaccines were not available. Furthermore, we defined reductions in COVID-19 infections and deaths as reductions in absolute numbers of COVID-19 infections and deaths and also reductions in the risk ratios of COVID-19 infections and deaths.

COVID-19 vaccines were not available in Kenya when the intervention took place. We therefore ensured all participants were masked and training activities and household visits in Siaya were conducted outside with universal mask wearing, and appropriate physical distancing.

We used the following algorithm to determine household members that were at a high risk for COVID-19 exposure and developing severe disease [28] (Table 1).

**Table 1:** Algorithm for determining household members that were at a high risk for COVID-19 exposure and developing severe COVID-19 disease.

Risk factors for COVID-19 exposure and developing severe COVID-19	Yes/No
Are you over 60 years old:	
Do you have hypertension:	
Do you have heart disease:	
Do you have diabetes:	
Do you have chronic respiratory disease:	
Do you have cancer:	
Have you been to any weddings, funerals, social gatherings or religious gatherings in the last 7 days:	
Have you had fever:	
Have you had a cough:	
Have you felt more tired than usual:	
Have you had any shortness of breath:	
Have you had any changes in taste or smell:	
Have you traveled to Asia, Europe, or the United states in the last 2 weeks? Have you traveled to Nairobi or Mombasa in the last 2 weeks?	

If household members presented with symptoms of COVID-19 with a recent history of possible exposure to COVID-19, isolation and testing was recommended to them.

In addition, we used the WHO SARS-CoV-2 contact definition as follows:

A SARS-CoV-2 contact is a person who has had any one of the following exposures to a probable or a confirmed case of SARS-CoV-2 infection: 1. face-to-face contact with a probable or confirmed case within 1 meter and for at least 15 minutes, or 2. direct physical contact with a probable or confirmed case, or 3. direct care for a patient with probable or confirmed COVID-19 disease without the use of recommended personal protective equipment (PPE) [29].

### Comparison non-intervention county—Kisii county

#### Comparison county

We compared Siaya county to Kisii county because both are rural; they are geographically distant (reducing the chance of contamination during the intervention period); and they have similar baseline health indicators in terms of age standardized mortality, life expectancy, and HIV deaths. In addition, both counties had similar critical care capacity: Kisii had 9 ICU beds and Siaya had 8 ICU beds. Table 2 summarizes baseline health and sociodemographic indicators in Siaya and Kisii counties.

**Table 2:** Baseline health and sociodemographic indicators in Siaya versus Kisii counties [30, 31].

Health and sociodemographic indicator	Siaya	Kisii
Population	993,183	1,266,860
Population density	393	957
Life expectancy	64.7	67
Baseline standardized mortality/ 100,000	1190	1140
HIV deaths/100,000	179	186
Fertility rate	4.2	3.7
Skilled birth attendance	97.8	97.7
Nutritional status of children, percent below—3 SD	7.1	9.3
HIV testing coverage	98.8	99.1
Education attainment women: % no education	1.9	0.9
Education attainment: men % no education	0.7	0.4
Literacy: women	33.7	48.3
Literacy: men	42.3	58.7
Teenage pregnancy	13.6	15.9
ANC percentage receiving skilled antenatal care	97.8	97.7
Delivery in health facilities	69.6	69.3
% delivered by a skilled provider	70.4	72.8
All basic vaccinations	79.3	84.6

*Siaya and Kisii counties have similar baseline health indicators in terms of age standardized mortality, life expectancy, and HIV deaths*.

In Kisii county, the standard national COVID-19 protocol was implemented. The standard national COVID-19 protocol emphasized the identification and preparation of isolation and treatment facilities at referral hospitals. It emphasized the capacity building of facility-based health workers. It required enhanced surveillance at all ports of entry and subnationally at county borders. It also emphasized the procurement of supplies, pharmaceuticals, and PPE for health facilities. The national government COVID-19 protocol did not include CHW training, equipping, and deployment.

### Data collection and analysis

i) We collected data on CHW household visits, COVID-19 infections, and COVID-19 deaths in Siaya and Kisii counties using the Kenya DHIS2 database. DHIS2 data collection protocols are the same across counties.

ii) We compared the number of CHW household visits in Siaya and Kisii counties. We measured risk ratios for COVID-19 infections and deaths in Siaya and Kisii counties. We organized data into contingency tables and divided the cumulative incidence of COVID-19 infections and COVID-19 deaths in Siaya by the cumulative incidence of COVID-19 infections and COVID-19 deaths in Kisii. In addition, we compared CHW skills in Siaya and Kisii counties based on digital records of vital signs at the household level.

#### Statistical analysis

Our goals for the statistical analysis were to determine effect sizes linked to the CHW intervention and to evaluate their significance. As previously described, the effect size is a quantitative summary measure obtained by comparing outcome measures between two or more groups [32]. Both dichotomous outcome measures (COVID-19 infections and deaths) and continuous outcome measures (number of households visited and number of vital signs measured at the household level) were recorded. To determine the effect sizes of our dichotomous data, we organized our data into 2x2 contingency tables and calculated risk ratios of COVID-19 infections and COVID-19 deaths in Siaya County compared to Kisii County. To further interpret risk ratios, we converted them to percentage change taking into consideration percentage differences between the 2 counties in COVID-19 infections and COVID-19 deaths for each month of the intervention as described by Ogallo et al. using the formula below. In this formula “i” represents a given month of the intervention, “yi” is the outcome at month i in the site where the intervention took place; and “y^i” is the outcome at the control site [33].


$$\mathrm{Percent}\;\mathrm{change}=\frac{1}{N}\;\sum_{i=1}^{N}\frac{\left(y_{i}-{\hat{y}}_{i}\right)}{{\hat{y}}_{i}}$$

We calculated confidence intervals to determine the significance of the risk ratios. We considered confidence intervals that excluded 1 and p-values less 0.05 to be statistically significant.

For continuous data (CHW household visits), we calculated the effect size by determining whether there were differences in the mean number of CHW household visits conducted in Siaya and Kisii counties. We determined if the differences in means were statistically significant using the Student T test. Table 3 summarizes our statistical analyses.

**Table 3:** Summary of statistical analyses performed.

Type of outcome measure	Outcome measure	Summary measure	Effect size measure	Significance
Dichotomous	COVID-19 infections	Proportion	Risk ratio	95% confidence interval
Dichotomous	COVID-19 deaths	Proportion	Risk ratio	95% confidence interval
Continuous	CHW household visits	Mean and standard deviation	Difference between 2 means	p-value using the Student T test
Continuous	Vital signs measurements at household level	Mean number of vital signs measurements per COVID-19 infection	Difference in the average number of vital signs	No household vital signs measurements completed in Kisii county

Our null hypothesis was that training, equipping and deploying CHWs is not associated with improved CHW skills and performance, and downstream reduced COVID-19 infections and deaths. We made the assumption that observations of COVID-19 infections and deaths in each county were independent. We used SPSS version 20.0 to conduct the statistical analyses.

## Results

*i) Training and equipping CHWs*:

In Siaya, 1359 CHWs were trained and equipped. No specific COVID-19 CHW training and equipping took place in Kisii County.

*ii) CHW skills following the intervention in Siaya*:

Following the CHW intervention in Siaya, Siaya CHWs conducted household visits and digitally reported vital signs. Table 4 summarizes the number of vital signs digitally reported by CHWs in Siaya (Table 4).

**Table 4:** CHW skills: Digital reports of vital signs at the household level.

Vital signs	Total number of Siaya CHW digitally reported vital signs for a total of 383 COVID-19 infections	Mean number of vital signs measurements per COVID-19 infection	Total number of digitally reported vital signs in Kisii for a total of 897 infections	Mean number of vital signs measurements in Kisii per COVID-19 infection
Oxygen saturation measurements	9266	24.2	0	0
Body temperature measurements	6541	17.1	0	0

### Siaya CHW digital reports of vital signs at the household level

The mean number of oxygen saturation measurements conducted in Siaya and the mean number of temperature measurements per COVID-19 infection are summarised above. In Kisii County, CHWs did not digitally report vital signs at the household level.

*iii) CHW activity: Monthly CHW household visits in Siaya versus Kisii counties*:

We found significant differences in the mean number of CHW household visits conducted in Siaya and Kisii Counties. The mean monthly number of CHW household visits was significantly higher in Siaya versus Kisii (the mean monthly CHW household visits in Siaya was 146,648.5, standard deviation 11,066.5 versus 42,644.5 in Kisii, standard deviation 899.5, p value = 0.01). The differences in the mean number of household visits conducted was statistically significant.

**Fig 3:**
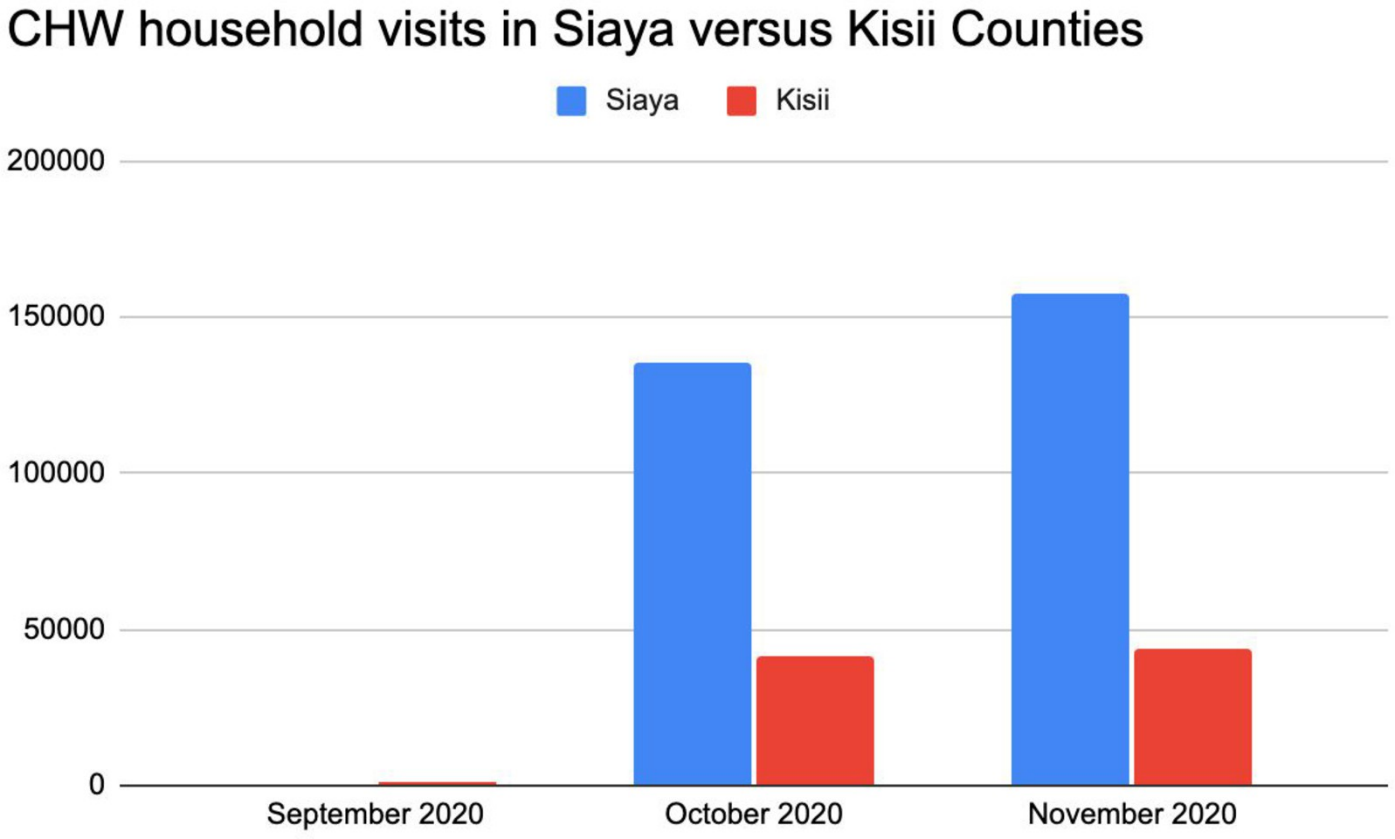
Monthly household visits in Siaya and Kisii counties.

Fig 3 summarizes differences in CHW household visits in Siaya and Kisii Counties (Fig 3).

### Differences in CHW household visits in Siaya and Kisii Counties

*iv) COVID-19 infections*:

We observed that increased CHW skills and increased CHW household visits were associated with fewer COVID-19 infections. Siaya County had a total of 383 infections from August to November 2020 and Kisii County had a total of 847 infections during the same period as shown in Table 5.

**Table 5:** 2x2 contingency table for COVID-19 infections in Siaya and Kisii counties.

COVID-19 infection	Yes	No	Total
**Siaya (CHW intervention)**	383	992,800	993,183
**Kisii (no CHW intervention)**	897	1,265,963	1,266,860
**Total**	1280	2,258,763	2,260,043

We found that the risk ratio for COVID-19 infections in Siaya was 0.54, 95% CI [0.48–0.61] implying a 46% reduction in the risk of COVID-19 infection in Siaya. Because the confidence interval excludes 1, we rejected the null hypothesis that there is no association between building the capacity of CHWs and a lower risk of COVID-19 infections in rural Western Kenya.

*v) COVID-19 deaths*:

We observed that increased CHW skills and increased CHW household visits with digital reporting of vital signs at the household level was associated with fewer COVID-19 deaths. Siaya County had a total of 7 deaths from August 2020 to November 2020; and Kisii County had 31 deaths during the same period as shown in Table 6.

**Table 6:** 2x2 contingency table for COVID-19 deaths in Siaya and Kisii counties.

COVID-19 deaths	Yes	No	Total
**Siaya (CHW intervention included)**	7	993,176	993,183
**Kisii (CHW intervention not included)**	31	1,266,829	1,266,860
**Total**	38	2,260,005	2,260,043

The risk ratio for COVID-19 deaths in Siaya was 0.29, 95% CI [0.13–0.65] a 71% reduction in risk, consistent with an effect of building the capacity of CHWs and lower risk of COVID-19 deaths in rural Western Kenya.

## Discussion

In this study, we found that training, equipping, and deploying CHWs in Siaya County was associated with increased CHW skills, increased CHW activity, and significantly lower COVID-19 infections and COVID-19 deaths compared with Kisii County that followed the Kenya national COVID-19 protocol but where no such training occurred. Following the addition of the CHW intervention to the standard national COVID-19 protocol, Siaya CHWs were skilled in capturing and digitally reporting vital signs at the household level; they conducted significantly more household visits than Kisii CHWs. The increased CHW skill and number of household visits were associated with reduced risk ratios of COVID-19 infections and COVID-19 deaths in Siaya. The likely mechanism explaining the effectiveness of rural CHWs in improving COVID-19 outcomes is that training and equipping CHWs led to increased CHW skills and CHW activity that in turn led to better COVID-19 case management at the household level with reduced COVID-19 infections and deaths.

*i) Training and equipping CHWs during the COVID-19 pandemic*:

A key component of the CHW intervention in Siaya was the training and the equipping of CHWs with pulse oximeters, thermometers, and KN95 masks. The ability to measure vital signs at the household level was particularly important in rural Western Kenya during this time, because COVID-19 testing capacity was limited [34]. Measuring vital signs at the household level likely contributed to the early identification of potential COVID-19 cases and the isolation of these cases by CHWs leading to the break in the community transmission of COVID-19 and fewer COVID-19 infections. Furthermore, the identification of COVID-19 cases at the household level likely increased the timely referral of severe cases to facilities with potentially life-saving oxygen capacity, probably contributing to significant reductions in COVID-19 deaths [35].

Other studies report interventions to train rural CHWs during the COVID-19 pandemic. Singh SS et al. trained 15 ’000 Ashas in the rural state of Bihar during the second COVID-19 wave in India [22]. Following this training, they found that > 80% of trained CHWs were satisfied with the training [22]. Kharel et al. trained 300 CHWs on COVID-19 in rural Nepal to increase their knowledge of COVID-19 [23]. Their preliminary results showed an increase in CHW knowledge of COVID-19 following their intervention. Another study from Uganda trained rural CHWs to help them identify, refer, and care for potential COVID-19 cases using a call center [25]. Similar to our study, these aforementioned studies show that it was feasible to train rural CHWs during the COVID-19 pandemic, and that the training of rural CHWs can lead to increased CHW capacity regarding a new disease. Furthermore, training rural CHWs during the COVID-19 pandemic was not only feasible in Asia, but also in SSA [22, 23, 25]. Our study reported additional outcome measures including CHW household visits and CHW measurement and digitally reporting vital signs following training.

*ii) CHW activity*:

Other studies have reported CHW visits during the COVID-19 pandemic. In a mixed methods study of rural indigenous communities in the Peruvian amazon, Reinders et al. document the resumption of household visits by two thirds of CHWs during the COVID-19 pandemic [36]. In this context, CHWs had little access to external support and training [36]. Similarly, in a mixed methods study Chengo et al. document CHW household visits in Kenya, Uganda, and Senegal during the COVID-19 pandemic [8]. Chengo et al. also highlight key challenges experienced by CHWs including the lack of protective gear, training, and reporting tools that were addressed by the intervention in Siaya County [8]. Addressing these challenges experienced by CHWs likely led to significant increases in CHW motivation and performance as shown by the significantly higher number of household visits conducted in Siaya; which subsequently led to the increased use of health services. According to Penchansky and Thomas’ theory of access, access to health services is optimized when the demand for services and the supply of services are maximized [37].

During a pandemic CHW activity will continue even in the absence of training and equipping CHWs. In our study, Kisii County reported CHW visits in the absence of additional CHW training and equipping. Salve et al. posit that CHWs tend to cope in the absence of adequate support and will continue to visit households during a pandemic; however, to maximize the performance and effectiveness of CHWs during a pandemic, our study shows that additional training and equipping is critical [7]. In rural Thailand, CHWs visited 14 million households from March to April 2020 following training according to Kaweenuttayanon 2021 et al, but our study establishes a stronger causal link between CHW training and CHW activity during the COVID-19 pandemic because of the comparative component in our research design [9].

*iii) CHW skills*:

In our study, we found that training and equipping CHWs was linked to increased CHW ability to measure and digitally report vital signs at the household level. Other studies have also reported CHW use of digital tools during the COVID-19 pandemic in Uganda, Ethiopia, and Mozambique [38]. To our knowledge, none of these studies used digital tools to report vital signs including oxygen saturation levels at the household level.

There is evidence to support the importance of oxygen level measurements for the early identification and management of hypoxemia during COVID-19 infections [35]. To our knowledge, no other study rolled out an intervention with CHWs measuring oxygen levels at the household level during the COVID-19 pandemic in rural SSA. There is critical care evidence that links the early identification and management of hypoxemia during COVID-19 infections with a reduction in COVID-19 deaths [35]. Sun et al provide useful algorithms for the identification and management of severe cases which includes the measurement of oxygen levels [35]. However, these oxygen saturation measurements were hospital based; in contrast to our experience in Siaya [35]. Our study shows that it is feasible for CHWs to measure and report vital signs including oxygen levels at the household level. CHW oxygen level measurements likely led to earlier referrals and management of severe COVID-19 in Siaya; and referrals to the right facility with oxygen capacity likely led to fewer COVID-19 deaths. Additionally, triaging of severe cases at the household level with CHW oxygen level measurements probably prevented hospitals from being overwhelmed with mild or moderate COVID-19 cases. In the rural context, this was a critically important consideration when human and material resources were even more scarce during an ongoing pandemic.

*iv) Effect of CHWs on COVID-19 infections*:

We also found that the increase in CHW skills and household visits in Siaya was linked to reduced risk ratios for COVID-19 infections in Siaya compared to Kisii. It is likely that because of these CHW efforts, COVID-19 cases were identified earlier and subsequently isolated earlier, thus reducing community transmission. The added ability to identify probable cases at the household level using vital signs was important because there were insufficient COVID-19 testing kits [30].

More CHW household visits in Siaya also meant increased likelihood of receiving health information which may have led to increased adherence to COVID-19 preventive measures. CHWs could have also influenced dimensions of the health belief model at the household level; specifically households’ perception of their susceptibility to COVID-19 infection, their perception of the severity of COVID-19 as a disease, their self-efficacy and perceived benefits and barriers to adopting certain health behaviors to prevent COVID-19 infection [39]. Kaweenuttayanon et al demonstrated reduced community COVID-19 transmission following the deployment of trained rural CHWs in March 2020 [9]. Within a 1 month of deploying trained rural CHWs, the daily numbers of new COVID-19 cases in Thailand dropped dramatically [9]. This study however was limited by the lack of mortality data.

*v) Effect of CHWs on COVID-19 deaths*:

In contrast to the Kaweenuttayanon et al experience, our study also showed an association between increased CHW skill and increased household visits and subsequent lower COVID-19 death rates in Siaya compared to Kisii. The most likely mechanism explaining this difference in mortality is that trained and equipped CHWs in Siaya were able to detect and manage COVID-19 cases earlier using their equipment. They likely were able to identify severe cases by measuring low oxygen levels and referring COVID-19 cases with low oxygen levels to facilities with oxygen capacity. In rural contexts, the capacity to identify severe cases and to refer them to a facility with oxygen capacity is important because distances to facilities are often far, means of transport are often unavailable, and road networks can be poor especially during the rainy season resulting in greater delays in reaching facility-based care compared to urban settings [5, 40]. Moreover, in rural health systems, health facilities are fewer in number with a limited health workforce, equipment, and supplies which can contribute to a delay in accessing care [5, 40]. Consequently, strengthening the diagnostic, monitoring, and case management capacity of CHWs in Siaya was particularly effective in mitigating the usual delays in access to care by bringing critical components of care to the household level which likely led to earlier referrals of severe COVID-19 cases, and referrals to health facilities with oxygen capacity averting a potential additional delay if patients are referred to a facility without oxygen capacity.

In summary, household contact with trained and equipped CHWs probably increased timely decision making and timely action to seek health services in facilities with oxygen capacity reducing delays in accessing lifesaving health services which led to fewer COVID-19 deaths in Siaya. Moreover, as previously mentioned training, equipping and deploying CHWs led to increased access to care and reduced transmission at the household level which also contributed to the fewer deaths observed in Siaya.

In Kenya, barriers to accessing care were heightened during the COVID-19 pandemic as a result of curfews and movement restrictions. Trained and equipped CHWs can reduce delays in making the decision to seek care, minimizing delays in reaching the hospital, and minimizing delays in receiving care [40]. To our knowledge, no other study links a rural CHW intervention with vital signs measurements at the household level to improved COVID-19 mortality outcomes.

Recently published Kenya DHS 2022 COVID-19 outcomes data validate our findings showing significantly lower COVID-19 mortality rates in Siaya compared to bordering counties in addition to Kisii County: Kakamega County was reported to have 55 COVID-19 deaths per 100’000 population; Kisumu County was reported to have 34 COVID-19 deaths per 100’000 population; Homa Bay County was reported to have 37 COVID-19 deaths per 100’000 population; and Busia County was reported to have 29 COVID-19 deaths per 100,000 population [41]. In the same survey, Kisii County was reported to have 34 COVID-19 deaths per 100,000 population and Siaya county 8 COVID-19 deaths per 100,000 population [41].

### Strengths of our study

Our study has several strengths. First, it has a comparative component in its research design which strengthens the causal link between trained, equipped, and deployed rural CHWs and significantly reduced COVID-19 infections and deaths. Second, this study demonstrates the feasibility of training, equipping, and deploying rural CHWs during the COVID-19 pandemic. This contributes to the implementation research evidence addressing the role of CHWs during pandemics. Our study showed its feasibility and contributed evidence towards its effectiveness. Third, our study has mortality data which prior studies addressing the role of rural CHWs in pandemics do not have. Fourth, our study addresses the rural population in SSA. To date, most studies addressing the role of CHWs during the COVID-19 pandemic are from Asia. Few COVID-19 studies emerged from rural SSA and there is a need for more evidence in this population because SSA is > 50% rural and Kenya is > 70% rural [4]. Lastly, we used routinely collected data which reduced potential biases including recall bias, reporting bias, and observer bias linked to the Siaya intervention. There was consistency in the variables collected across counties using the same methodology across counties.

### Limitations of our study

There were several limitations to our study. First, we compared Siaya to a county with similar characteristics; however there could be persistent confounding factors. As shown in Table 2, health indicators were similar in both counties. However, there were sociodemographic differences: the population density was higher in Kisii compared to Siaya which could lead to a higher risk of COVID-19 infection in Kisii. However, the evidence supporting the link between increased population density and increased respiratory disease transmission remains uncertain. In a systematic review of 21 studies by Zhang X et al, there was no consistent association between increased population density and increased respiratory disease transmission including COVID-19 [42]. Hamidi et al found that the connectivity of a location was a more important predictor of COVID-19 spread rather than population density [43]. Siaya being in close proximity with Busia, a county bordering Uganda, and Kisumu, an urban county with an airport, is more connected than Kisii, and would therefore be expected to have more COVID-19 infections. We observed the opposite in our study and adjusting for this confounding factor could potentially have demonstrated a greater effect measure associated with building the capacity of CHWs and reducing the risk of COVID-19 infections and deaths. Moreover, education attainment was higher in Kisii compared to Siaya. Increased education level of a population is often linked to better health literacy and therefore reduced disease transmission and death. Yoshikawa et al found that educational attainment was associated with a lower risk of severe COVID-19 disease [44]. Similarly, Gomes da Silva et al found that increased educational attainment was associated with increased health literacy about COVID-19 in Portugal [45]. Adjusting for a lower education attainment in Siaya could potentially have resulted in a greater effect measure being observed in our study. To address confounding factors, we considered conducting regression analyses including the Poisson regression as described by Ogallo W et al.; however, our data was limited by the fact that we did not have individual level data [33].

Additional limitations to our study could be delays in capturing and reporting COVID-19 infections; however, reporting mandated by the National Emergency Response Committee on Coronavirus ensured daily reporting of new COVID-19 cases and deaths by all counties. In addition, there could have been differences in oxygen use in both counties and not measuring differences in oxygen use is a limitation of our study. However, in the Kenyan context, the major rate limiting factor across all counties in Kenya was not the amount of oxygen available but the ability to deliver oxygen using ventilators; the number of ventilators was in the single digits in both counties (8 in Siaya and 9 in Kisii). Furthermore, transport delays may have played a role in the differences in mortality; however, we observed better COVID-19 outcomes in Siaya where distances travelled to reach health facilities are greater. This finding further supports the effectiveness of CHWs in reducing barriers to care including distance to facilities.

Lastly, we used DHIS2 data to conduct our comparative analysis. DHIS2 data includes data from public, non-governmental, and faith-based health facilities. It does not include data from private health facilities. Therefore, our findings may not apply to populations with significant use of private facilities or to urban populations. Large scale cluster randomised trials could provide more robust evidence but are resource intensive and difficult to launch rapidly in pandemics. They would need to be planned in advance with an agreed protocol that could be adapted to the prevailing circumstances.

### Policy implications

Disease outbreaks and pandemics remain a significant threat to rural populations that are particularly vulnerable because they are under-resourced in terms of infrastructure, equipment, and a health workforce. Our study showed that training, equipping, and deploying CHWs can strengthen pandemic preparedness and response and lead to fewer COVID-19 infections and deaths compared to standard measures. In a global context of continued disparities in access to COVID-19 therapeutics and vaccines, a pattern repeated at all stages of the COVID-19 pandemic, defining interventions that would leverage assets already present in rural health systems, including CHWs is crucial for robust current and future pandemic preparedness and response efforts [46]. The policy implications of our results from rural SSA are that building a community workforce is a critical component of pandemic preparedness and response especially in under-resourced health systems including rural health systems. Policymakers with significant rural populations particularly in SSA should consider investing in CHW capacity building as part of current and future pandemic preparedness and response strategies to save lives.

## Conclusion

In conclusion, our study showed that training, equipping, and deploying rural CHWs was associated with significantly lower risks of COVID-19 infections and deaths in rural Western Kenya. Regions with significant rural populations should strongly consider training, equipping, and deploying rural CHWs to strengthen their pandemic preparedness and response efforts to save lives.

## Supporting information

S1 Checklist Completed Inclusivity-in-global-research-questionnaire August 14 2023. (PDF)
